# Blurred vision due to choroidal metastasis as the first manifestation of lung cancer: A case report

**DOI:** 10.1186/1477-7819-8-2

**Published:** 2010-01-08

**Authors:** Christos Asteriou, Dimitrios Konstantinou, Athanassios Kleontas, Dimitrios Paliouras, Georgios Samanidis, Fani Papadopoulou, Nikolaos Barbetakis

**Affiliations:** 1 Cardiothoracic Surgery Department, Theagenio Cancer Hospital, Al Symeonidi 2, Thessaloniki, Greece, 54007; 2 2nd Department of Chemotherapy, Theagenio Cancer Hospital, Al. Symeonidi 2, Thessaloniki, Greece, 54007

## Abstract

**Background:**

Reduction in visual acuity combined with blurred vision is rarely the first sign of lung cancer and very few cases have been announced globally.

**Case presentation:**

A case of a 46-year-old man who admitted with blurred vision is presented. His medical history, apart from a mild gastritis under treatment was negative. Ocular examination revealed a decrease in visual acuity due to a choroidal tumor. Further image body scans demonstrated a right lung lesion with dissemination to other organs. Diagnosis of a non-small cell lung cancer established after a VATS biopsy carried out.

**Conclusion:**

Blurred vision due to choroidal metastasis as the primary symptom of lung cancer is very uncommon. A great index of suspicion is essential when a choroidal lesion appears.

## Background

The incidence of ocular metastases from lung cancer is reported to be 2-7% according to the international literature [1,2]. The majority of cases involves end-stage patients. Choroidal metastases are metastatic lesions to the choroid layer of the eye. Decrease in optical acuity as the initial manifestation of lung cancer is infrequent.

## Case presentation

**Figure 1:**
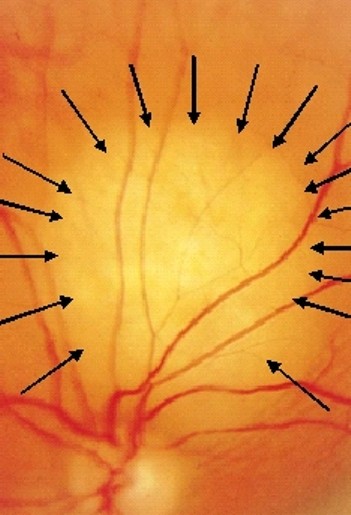
**Left ocular fundus photo composite shows superior solitary choroidal metastase (black arrows)**.

**Figure 2:**
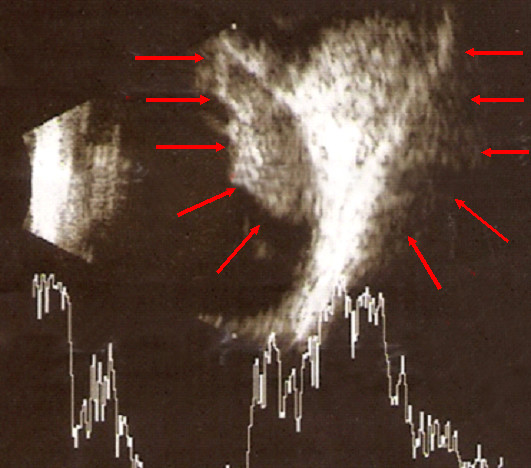
**Transverse B-scan ultrasound of superior posterior pole, left eye, shows choroidal mass (red arrows)**.

A 46-year-old man noticed, three months ago, that for the past two weeks his left eye suffered from reduction in vision with a concomitant blurredness. He had no medical history apart from a mild gastritis under treatment. He was a heavy smoker (one and a half pack of cigarettes for the last 27 years), while he was a social drinker. Examination showed visual acuity of 2/20 in the left eye and normal in the right eye. Fundus examination revealed the presence of a choroidal tumor located superior to the optic nerve measuring 921 mm (Fig 1). An ultrasonographic evaluation of the eye demonstrated that the tumor had a height of 1.8 mm (Fig 2). Computed tomography of the chest, abdomen and brain were performed. A central lesion of the right upper lobe of the lung was detected. Both, a small pleural effusion and several mediastinal enlarged lymph nodes were accompanying the aforementioned lesion. Moreover, small, bilateral adrenal masses were also present, while three brain metastases appeared. A bone scanning revealed multiple lesions.

**Figure 3:**
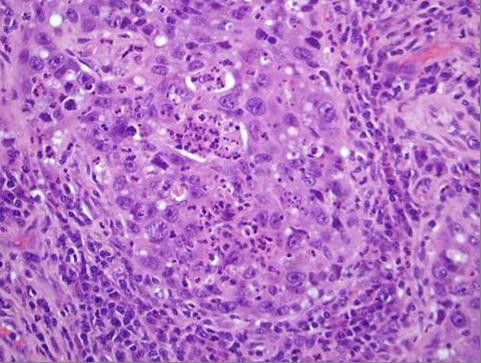
**Squamous cell carcinoma of lung. Histopathological view (H-E × 200)**.

A transthoracic needle biopsy of the right upper lobe lung mass could not provide enough material to establish an accurate histopathological diagnosis. The patient experienced a Video Assisted Thoracic Surgical biopsy and the diagnosis of a low-grade squamous cell carcinoma was established (Fig 3). He was discharged after 5 days. The patient is currently receiving chemotherapy and radiotherapy.

## Discussion

The highly vascular uveal tract is the most common part of the eye involved by metastases. Within the uvea, the choroid (88%) is the most commonly affected site followed by the iris (9%) and ciliary body (2%) [3]. Breast cancer seems to be the most frequent type of cancer giving intraocular metastases. The incidence for the breast cancer is reported to be 37-41%, while lung cancer is considered to be responsible for no more than 7% of choroidal metastases [4,5]. It is generally considered that these kind of metastatic lesions occur at the final stage of the disease, where the mean survival is not expected to be more than 6 months and the majority of the patients already suffer from the typical lung cancer symptoms [6]. The reason for this unusual site to be target for secondary metastases from lung cancer is generally unknown. It is, however, speculated that its high vascularity may consist a reasonable explanation [7]. The diagnosis of ocular metastases is based primarily on clinical findings supplemented by imaging studies. The diagnostic procedures include ultrasonography, fluorescein angiography, computed tomography/MRI, fine-needle aspiration, or wedge biopsy. Brain imaging is useful before initiation of radiotherapy to assist in treatment planning. It is reported that 22% of patients diagnosed with choroidal metastasis had a concurrent diagnosis of central nervous system metastasis [8]. Differential diagnosis includes primary choroidal melanomas, benign lesions such as haemangioma, and inflammatory granulomas.

Decrease in vision and blurredness as the initial manifestation of lung carcinoma is therefore very rare. A review of the literature brought to light 12 cases of lung cancer patients suffering from choroidal metastases as the primary clinical sign. In all cases, the diagnosis of the choroidal metastases declares final stage disease and the dissemination seems to be almost certain. Prognosis is poor and life expectancy short.

Treatment for ocular metastases is palliative because the presence of such metastases suggests hematogenous spread of cancer. In line with this, the aims for treatment are to maximize quality of life, and restore or preserve vision. This may be achieved with either radiotherapy or chemotherapy. Surgery has not played an important role other than diagnostic biopsy, as surgery carries great potential morbidity and often there is no need for tumor debulking.

## Conclusions

In conclusion, when a choroidal lesion is discovered, the differential diagnosis should include secondary metastases due to malignant tumors originated from distal organs.

## Consent

Written informed consent was obtained from the patient for publication of this case report and accompanying images. A copy of the written consent is available for review by the Editor-in-Chief of this journal.

## Competing interests

The authors declare that they have no competing interests.

## Authors' contributions

CA, DK, AK, DP, GS, and FP took part in the care of the patient and contributed equally in carrying out the medical literature search and preparation of the manuscript. NB participated in the care of the patient and had the supervision of this report. All authors approved the final manuscript.
